# Aquaporin Modulation by Cations, a Review

**DOI:** 10.3390/cimb46080470

**Published:** 2024-07-24

**Authors:** Robin Mom, Vincent Mocquet, Daniel Auguin, Stéphane Réty

**Affiliations:** 1 Laboratoire de Biologie et Modelisation de la Cellule, Ecole Normale Superieure de Lyon, CNRS, UMR 5239, Inserm, U1293, Universite Claude Bernard Lyon 1, 46 allee d’Italie, F-69364 Lyon, France; vincent.mocquet@ens-lyon.fr; 2 Laboratoire de Physiologie, Ecologie et Environnement (P2E), UPRES EA 1207/USC INRAE-1328, UFR Sciences et Techniques, Université d’Orléans, F-45067 Orléans, France; auguin@univ-orleans.fr

**Keywords:** AQP, cation, regulations, calcium, mercury

## Abstract

Aquaporins (AQPs) are transmembrane channels initially discovered for their role in water flux facilitation through biological membranes. Over the years, a much more complex and subtle picture of these channels appeared, highlighting many other solutes accommodated by AQPs and a dense regulatory network finely tuning cell membranes’ water permeability. At the intersection between several transduction pathways (e.g., cell volume regulation, calcium signaling, potassium cycling, etc.), this wide and ancient protein family is considered an important therapeutic target for cancer treatment and many other pathophysiologies. However, a precise and isoform-specific modulation of these channels function is still challenging. Among the modulators of AQPs functions, cations have been shown to play a significant contribution, starting with mercury being historically associated with the inhibition of AQPs since their discovery. While the comprehension of AQPs modulation by cations has improved, a unifying molecular mechanism integrating all current knowledge is still lacking. In an effort to extract general trends, we reviewed all known modulations of AQPs by cations to capture a first glimpse of this regulatory network. We paid particular attention to the associated molecular mechanisms and pinpointed the residues involved in cation binding and in conformational changes tied up to the modulation of the channel function.

## 1. Introduction

Aquaporins (AQPs) constitute a wide and ancient family of transmembrane channels dedicated to the facilitation of water passage across cells membranes [1,2,3]. They also accommodate other small polar solutes such as glycerol (channeled by aquaglyceroporines: AQGP), ammonia (channeled by aqua-ammoniaporines), H_2_O_2_ or even anions [4,5,6,7]. In humans, 13 AQPs are found and are distributed in different tissues and cell types, depending on their substrate selectivity and on their regulations [1,8]. Because AQPs constitute the main pathway for cells to dynamically regulate their membranes water permeability, they are central in the regulation of cell homeostasis (through the regulation of water and ions concentrations), cell volume regulations (regulatory volume decrease [9,10,11,12], cell proliferation [13] and cell motility [14]) and trans-cellular water fluxes [8] (e.g., water reabsorption in kidney collecting duct [15], lymph fluxes in the inner ear [16], all types of secretions in dedicated glands [17], etc.). Additionally, other roles involving the other substrates accommodated by AQPs have been described [7,18,19]. Finally, it should be kept in mind that all of the subtleties of the various possible regulations regarding AQPs have not yet been elucidated [20,21]. Obviously, this wide spectrum of functions makes AQP dysregulations a common feature of many different pathophysiologies [22,23], especially in cancer cell development and proliferation [24,25,26].

**Figure 1:**
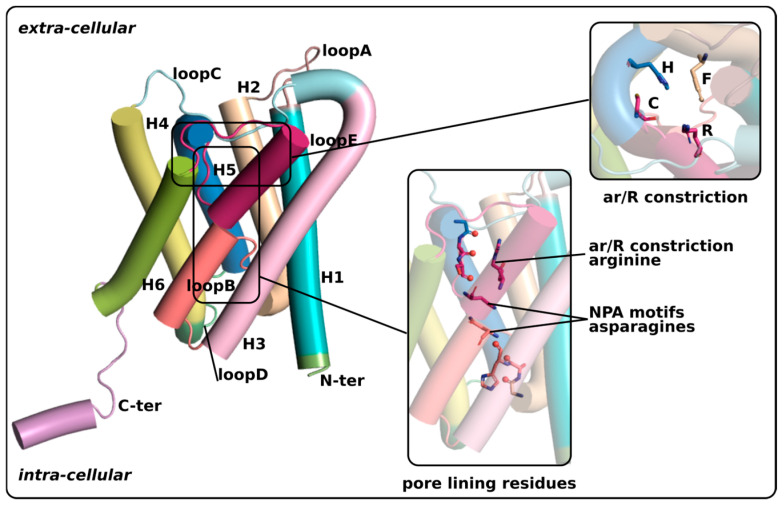
Schematic representation of an AQP. The X-ray structure of human AQP2 was used to make this representation. The two main constrictions within the conducting pore of AQPs are the NPA motifs region and the ar/R constriction. Waters inside the pore (not represented) form single file continuum because of the hydrophobic nature of many pore lining residues. Only a few residues in loop B and loop E have their backbone oxygen (represented by a red sphere) oriented toward the pore lumen, creating punctual polar relays as successive interaction sites for water across the pore. In addition, the polar groups of constitutive sidechains, including NPA motifs asparagines and ar/R constriction polar or charged residues, complete this suite.

AQPs are found in tetrameric assemblies, with each of the four subunits being a functional pore [27]. Each subunit is made out of six transmembrane alpha helices (H1–H6) that provide the framework for the channel (Figure 1). The angle at which the helices are orientated creates two funnel-shaped vestibules (intra-cellular and extra-cellular) connected together by the conducting pore. This conducting pore is characterized by several water-interacting sites able to complement the hydrophilic nature of water molecules. These sites are mainly constituted by the backbone carbonyl oxygens of loop E of the extra-cellular vestibule and loop B of the intra-cellular vestibule [27]. Both of these two loops contain small alpha helices (HE and HB, respectively) that meet at the center of the subunit. Each of the small helices possesses an NPA motif (for an asparagine–proline–alanine motif) that meets the other’s at the center of the pore. At this location, the dipole moments of HB and HE create a positive electrostatic field. This particularity has been shown to be responsible for proton exclusion from the pore and is also a water-interacting site [27,28]. The rest of the conducting pore is hydrophobic, hence forcing water molecules to form a single-file hydrogen network continuum between the ends of the two vestibules. Finally, in the extra-cellular vestibule, three or four residues make the aromatic/arginine (ar/R) constriction, which corresponds to the narrowest part of the channel and from which originates substrate selectivity between classes of aquaporins [27,29]. On top of this structural unit core, flexible loops and N-terminal and C-terminal extremities protrude into the cytoplasm (loop D and loop B, N-ter and C-ter) and in the extra-cellular compartment (loop A, loop E and loop C). The sequence variability is higher in these flexible loops and extremities [1], which are finely involved in the regulation of the core function of AQPs [30]. 

AQPs are regulated by many different types of molecules, including protons [31,32], small circulating molecules [33], membrane components [34] or other proteins [35]. In the current review, we intend to summarize the current knowledge about AQP regulation by cations, describe the associated molecular mechanisms and pinpoint the key residues involved.

## 2. Modulation by Alkaline Earth Metal Cations

### 2.1. Calcium Ions

Calcium ions are known as one of the most ubiquitous secondary messengers in living cells, transducing many types of signals depending on their concentration, sub-cellular localization [36], route of entry in the cytoplasm [37] and pattern of release (amplitude and frequency) [38]. Many proteins are regulated by calcium, with the most studied family of calcium sensors being the calmodulin family [39]. Calmodulins (CaMs) constitute a highly conserved family of proteins involved in calcium sensing and signaling. Upon calcium binding, changes in conformation induce the opening of the E-F hand motifs, exposing hydrophobic surfaces of the two alpha helices and allowing binding to proteins regulated by calmodulins [39,40]. 

**Figure 2:**
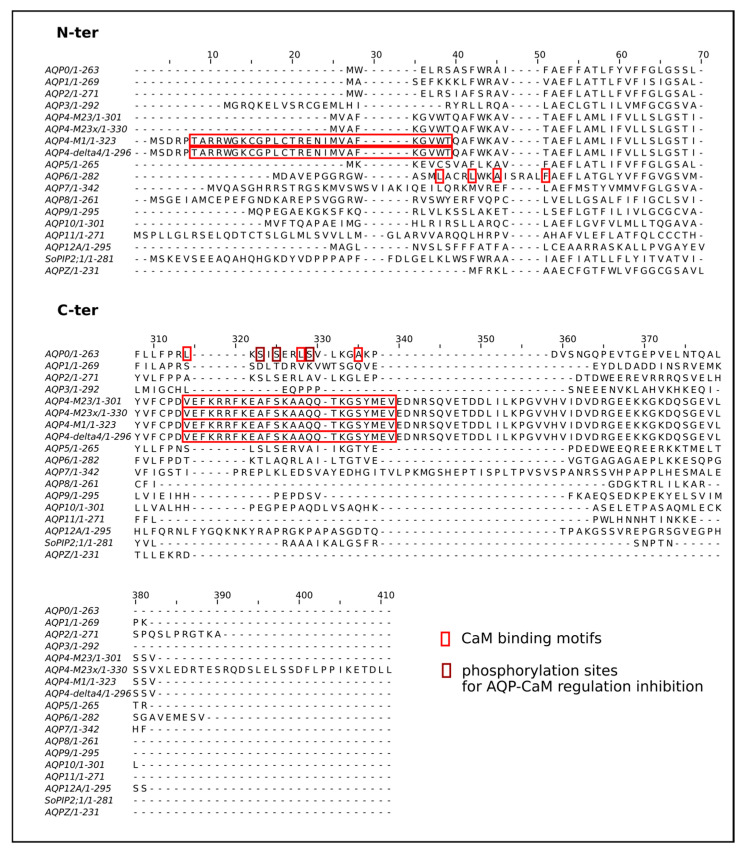
AQP regulation by calmodulin. Multiple sequence alignment of human AQPs, spinach *So*PIP2;1 and *E. coli* AQPZ. CaM binding motifs or fragments used for binding assays extracted from [41,48,49] are reported on the corresponding human sequences. Phosphorylation sites for AQP-CaM regulation inhibition in human AQP0 are extracted from [46].

The first type of regulation involving calcium is indirect and is mediated through the activation of calmodulin by calcium. This regulation was mostly studied for the eye AQP0 [32,41,42,43,44,45,46,47]; however, other studies point to other AQPs as being regulated by calmodulin and calcium as well, such as AQP6 [48], AQP4 [49] or AQP2 [50,51,52,53]. With the exception of AQP2, whose function is regulated by calmodulin through the modulation of its cellular trafficking [50,51,52,53], AQPs (AQP0, AQP4 and AQP6) seem to interact directly with calmodulins [41,44,45,48,49] at a 1-8-14 like motif located on their C-terminal or N-terminal extremities (Figure 2) [41,48,49]. The fixation of calmodulin on the AQP0 tetramer inhibits its water permeability. Upon binding, conformational changes induce an increase in monomer cooperativity associated with a reduction of water pore size at the two constrictions of the channel (R187 and Y149) and near Y23 [45,54,55]. Arginine 156 located on intra-cellular loop D of ovine AQP0 seems to play a key role in this allosteric mechanism [55]. Three phosphorylation sites located at the C-terminal extremities have also been implicated in the regulation of calmodulin binding to human AQP0. Upon the phosphorylation of S229 or S235, calmodulin interaction is prevented (Figure 2). When S231 is phosphorylated, however, calmodulin can bind to AQP0, but the water permeability is not impacted [46]. 

**Figure 3:**
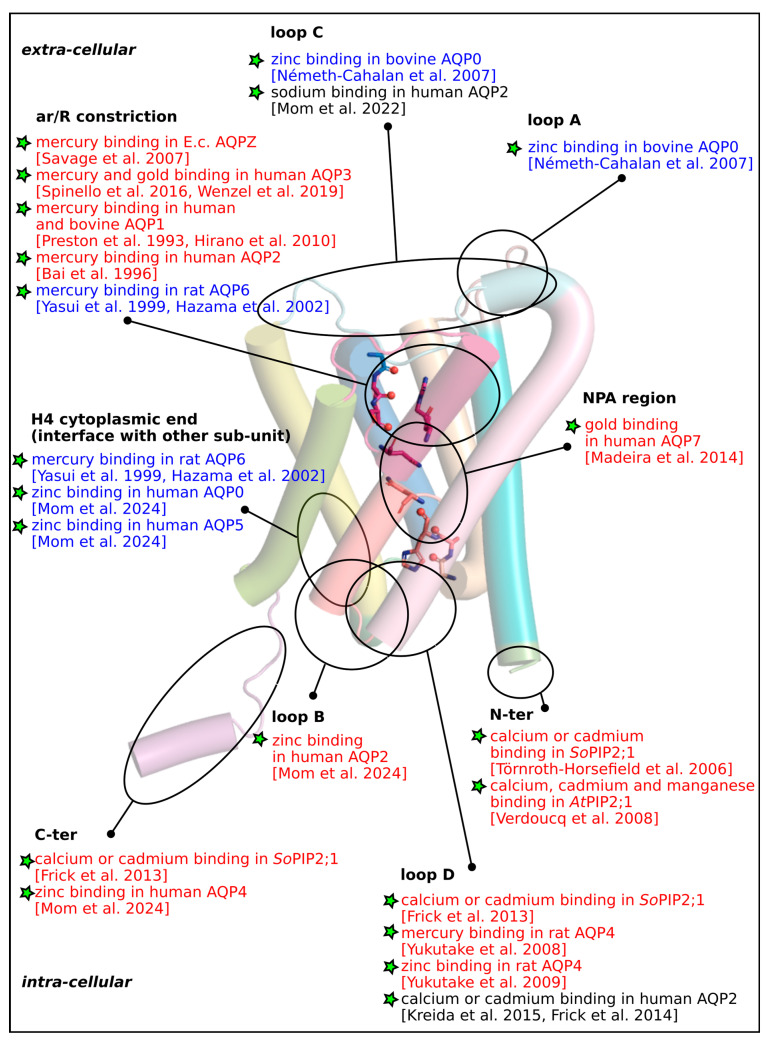
Cation binding sites in AQPs. The cations binding sites mentioned in the current review are indicated on a schematic representation of an archetypal AQP (made from X-ray structure of human AQP2). The reference scientific paper for each binding site is indicated under brackets: Savage et al. 2007 [82]; Spinello et al. 2016 [86]; Wenzel et al. 2019 [114]; Preston et al. 1993 [80]; Hirano et al. 2010 [88]; Bai et al. 1996 [87]; Yasui et al 1999 [85]; Hazama et al. 2002 [90]; Mom et al. 2022 [157]; Mom et al. 2024 [102]; Frick et al. 2013 [60]; Frick et al. 2014 [63]; Yukutake et al. 2008 [83]; Yukutake et al. 2009 [105]; Kreida et al. 2015 [56]; Törnroth-Horsefield et al. 2006 [31]; Verdoucq et al. 2008 [62]; Madeira et al. 2014 [117]; Németh-Cahalan et al. 2007 [101]. Cation binding can be associated with AQP activation (legend written in blue), inhibition (legend written in red) or unknown event (legend written in black).

Additionally, calcium has been directly involved in plant AQP gating [31,56]. More precisely, the experimental X-ray structure of spinach AQP *So*PIP2;1 revealed cadmium cations bound to the intra-cellular N-terminal residues E31 and D28 (Figure 3). In plants, strict AQPs (PIP, for plasma membrane intrinsic protein) possess a long intra-cellular loop D. This flexible loop can switch from an open to a closed conformation in which a few residues create a hydrophobic patch preventing water passage through the intra-cellular vestibule of the AQP. According to the authors, this cation participates in the locking of the closed conformation by joining loop D to the facing N-terminal extremity through a hydrogen bond/salt bridge network [31]. Two residues of loop D (R190 and D191) are also crucial to maintaining the loop in a closed conformation [31,57]. According to the authors that solved the closed conformation of *So*PIP2;1, the closing of the channel by loop D would be triggered and directed by the protonation of H193 [31]. A phosphomimicking mutant study investigated the role of phosphorylation of S115, S188 and S274 [58]. Serine 115 is situated close to the divalent cation binding site, and its mutation into a glutamate disrupted the hydrogen bond and salt bridge lock [31,58]. The serine 274 to glutamate mutant was shown to disorder the C-terminal extremity, but its exact role is still misunderstood [58]. Finally, S188 phosphorylation was associated with the opening of the pore through conformational rearrangements of loop D, disrupting R190 and D191 anchoring to the N-terminal residues [58,59]. As mentioned previously, no direct observation of calcium bound to *So*PIP2;1 was made (i.e., cadmium was observed in interaction with the AQP) however, the authors searched for similar structural motifs and found 13 other experimental structure entries containing calcium ions [31]. Another cadmium ion was found in another structure of *So*PIP2;1, and it interacts directly with T183 and A267 [60]. Following the same idea as for the D28–E31 first cadmium binding site, the authors postulated this second site to correspond to a lower affinity binding site for calcium [60]. Moreover, the phosphorylation of a spinach AQP at S274 was shown to be calcium-dependent in vitro [61], comforting the reliability of this regulation of spinach AQPs by calcium. In another study, the effects of calcium and other cations were tested on *Arabidopsis thaliana* PIP2;1 (*At*PIP2;1) reconstituted in proteoliposomes [62]. The impact of the cations on the water permeability of *At*PIP2;1 was estimated through the stopped-flow light scattering of the proteoliposomes. Calcium was associated with the strongest inhibition of *At*PIP2;1 after cadmium. The mutation of residues involved in plant AQP gating described for *So*PIP2;1 (D28, E31 and H193) to alanine diminished (for D28A mutant in *At*PIP2;1) or completely canceled (for E31A and H199A mutants in *At*PIP2;1) the sensitivity to calcium of *At*PIP2;1. Interestingly, the mutation of another residue located on loop B (R124 in *At*PIP2;1) also largely reduced *At*PIP2;1 calcium sensitivity [62]. 

Similarly to *So*PIP2;1, the human AQP2 experimental structure also contained cadmium ions in interaction with the intra-cellular surface of the tetramer [63]. In the structure, the cation is stabilized by Q57 and E155 [63]. Next to Q57, S148 is located within a casein kinase II consensus site [56]. Hence, in a similar manner as in *So*PIP2;1 regulation, the phosphorylation of S148 could disrupt the salt bridge network stabilizing the cation. Interestingly, a phosphomimicking mutation of S148 induced AQP2 retention in the Endoplasmic reticulum [64]. Moreover, the mutation of Q57 or A147 (which is close to the cation fixation site) has been identified in patients suffering from nephrogenic diabetes insipidus, a disorder caused by the failure to address AQP2 to the apical membrane [65]. Even though the exact mechanism is still unknown, it is clear that calcium and calmodulin play a significant role in the regulation of AQP2 trafficking [50,51,52,53]. 

### 2.2. Magnesium Ions

Magnesium is the major intra-cellular divalent cation and plays essential physiological roles (such as in the normal function of the cardiovascular system) [66]. It is involved in proteins and nucleic acid synthesis and is a co-factor of many enzymes. Most of the magnesium in the human body is found in bones (60%), of which about 30% is exchangeable. It is otherwise mainly present within cells (20% in skeletal muscle and 19% in soft tissues), with only 1% in extra-cellular fluids [66].

Several studies have shown that magnesium treatment (magnesium sulfate, magnesium acetate) was associated with a significant increase in human AQP3 transcript levels [67,68,69]. Moreover, all three studies also observed a significant increase in adenylate cyclase (AC) and protein kinase A (PKA) activity and an increase in phosphorylation of the cAMP response element-binding protein (CREB). Hence, intra-cellular magnesium ion increases seem to activate AC, which in turn triggers CREB phosphorylation through the activation of PKA, eventually promoting AQP3 gene transcription [67,68,69]. 

Other studies have highlighted a link between magnesium treatment and AQP4 expression. Studies led on the central nervous system tissues of rats have highlighted a significant decrease of AQP4 proteins through immunohistochemistry after treatment with magnesium sulfate [70,71]. However, another work studying the impact of magnesium sulfate on children with “hand, foot, and mouth” disease found a significant decrease of AQP4 in the serum but not in the cerebrospinal fluid after the magnesium sulfate treatment of patients [72]. Another study led on pregnant rats with acute hypertension indicated a significant reduction of blood–brain barrier permeability by magnesium sulfate treatment in the posterior cerebrum but not in the anterior cerebrum. However, no effect was observed on AQP4 expression [73]. Hence, the exact effect of magnesium sulfate on AQP4 expression and brain water homeostasis is still unclear. Moreover, AQP3 is also expressed in the brain [74]; hence, the physiological effect of magnesium sulfate on water balance observed in brain diseases could also be partly explained by the modulation of AQP3 transcripts level.

## 3. Transition Metals

### 3.1. Mercury Ions

Mercury ions are historically associated with the discovery and characterization of AQPs [75]. Initially, the discovery of red blood cell membranes’ water permeability inhibition by organic mercurials [76] allowed for later AQP isolation [77], cloning [3,78], membrane transport characterization [79] and mercury sensitivity mutational analysis [80,81]. Mercurial compounds are known to inhibit most AQPs [80,82,83,84], with the notable exception of human AQP6 [85] and spinach *So*PIP2;1 [60], for which mercury ions induce an increase in their water permeability and the activation of ion conductance in AQP6. 

The molecular mechanism associated with AQPs’ inhibition by mercury is due to its high affinity with the thiol group found in cysteine residues [82]. Many AQPs display cysteine in their ar/R constriction. Hence, in these cases, mercury binding was hypothesized to directly block the pore sterically [82]. However, depending on where the pore lining cysteine is located, the sensitivity to mercury can be increased. A study of *E. coli* AQPZ wild type and AQPZ mutants’ (pore-lining residues T183 and L170 mutated to cysteine) water permeability reconstituted in proteoliposomes coupled to the resolution of their 3D structures through X-ray crystallography highlighted this change in mercury sensitivity [82]. In wild-type AQPZ, the pore lining cysteine (C20) is part of the ar/R constriction and is located on helix 1. This wild-type AQPZ corresponded to the smallest inhibition by mercury [82]. The aquaglyceroporine AQP3 bears a cysteine at its ar/R constriction on the first alpha helix as well (C40). Another study showed through molecular dynamics that mercury binding to C40 inhibited water and glycerol passage because of small conformational changes in the residues of the ar/R constriction. More precisely, conformational changes of the sidechains of F63 and R218 obstructed the pore [86]. One of the AQPZ mutants corresponded to a cysteine replacing another residue of the same ar/R constriction (T183), which also mimicked the location of cysteine in human AQP1 (C189 in human AQP1) and which is located on loop B. Cysteine in position 183 increased the mercury sensitivity of AQPZ [82]. Moreover, no conformational changes could be observed in the AQPZ-T183C mutant structure when compared to the wild type [82]. Other site-directed mutagenesis studies demonstrated inhibition of AQP1 [80] and AQP2 [87] water permeability by mercury associated with the T183 corresponding cysteine (C189 and C181 in human AQP1 and human AQP2, respectively). In AQP1, however, in another molecular dynamics study, the authors observed small local conformational changes triggered by mercury fixation [88]. In this study, not only steric effects but also the repositioning of loop B backbone explained the inhibition. Indeed, loop B backbone carbonyl oxygens are water interaction sites; hence, their reorientation out of the pore created a hydrophobic patch locally [88]. The AQPZ mutant associated with the highest sensitivity to mercury had the pore-lining leucine 170 replaced by cysteine [82]. This leucine is located at the center of the pore facing the NPA asparagines and corresponds to a position in the pore that was predicted by the authors to maximize steric inhibition upon mercury binding [82]. A molecular dynamics study of this mutant inhibition by mercury revealed that steric inhibition of water passage could be explained by the re-organization of water molecules around mercury cation. According to the study, five to six molecules of water interacted strongly with mercury and were responsible for pore occlusion [89].

However, with more AQPs being studied for their modulation by mercury, it became clear that other molecular mechanisms were involved. AQP4 was initially considered a mercury-insensitive AQP only to later observe mercury inhibition when reconstituted into proteoliposomes [83]. This can be explained by the bi-directional orientation of AQP4 tetramers in proteoliposomes and by the fixation of mercury ions to an intra-cellular cysteine. Indeed, through the site-directed mutagenesis of rat AQP4 cysteines, the authors pinpointed intra-cellular cysteine 178 located on loop D as responsible for the transduction of AQP4 inhibition by mercury [83]. Contrarily to the previous situations, C178 is situated far from the ar/R constriction, and the Van der Waals radius of mercury is not large enough to occlude the pore at this location (i.e., at the entrance of the intra-cellular vestibule). This implies that other molecular mechanisms besides a simple steric blockage of the pore are at work to explain mercury inhibition of AQP4. The authors postulated that the gating of AQP4 through similar conformational changes of loop D as described for plant AQPs could be triggered by mercury fixation (see calcium section) [31,83]. Rat intra-cellular AQP6 water permeability increases and ion channeling activation have been shown to be triggered by mercury ions binding to cysteines 190 and 155 [85,90]. The mutation of C190 to alanine or C155 to alanine resulted in a 50% loss in mercury-inducible water and ions conductance, while the double mutation exhibited negligible mercury activation [85,90]. Similarly to AQP1, C190 is located in the ar/R constriction on the water-interacting sites bearing loop B, while C155 is positioned on the intra-cellular surface at the interface between two sub-units. Electrophysiological measurements coupled to site-directed mutagenesis revealed that changes in water permeability correlated with equivalent changes in ions conductance, indicating both water and ions were very likely to cross AQP6 through the same monomeric conducting pore [85,90]. From molecular dynamics studies, it appeared that the modulation of water flux upon mercury fixation in AQP6 was eventually integrated through the position of the ar/R constriction arginine and through other pore-lining residue methionine 160 (of rat AQP6) sidechains within the pore lumen [91,92]. The exact way in which mercury binding to intra-cellular cysteine 155 resulted in these allosteric modulations of pore-lining residues sidechain position within the pore lumen is still unclear. Finally, in plant AQPs, the modulation of water permeability is even more complex. Except for two intra-cellular AQPs of Arabidopsis thaliana (located in the tonoplast membrane) [93], no clear link could be found between cysteine residues and the transduction of mercury modulation of the water permeability [60,62,94]. In one study led on spinach AQP *So*PIP2;1, none of the cysteine to serine mutants differed significantly from the wild type in terms of mercury modulation. The water permeability of the wild type and of all of the cysteine mutants were similarly significantly increased by mercury [60]. From this study, it appears that mercury can hence impact AQPs’ function through yet another molecular mechanism not related to cysteines. The authors postulated a putative indirect action of the cation through the modulation of phospholipids’ bilayer fluidity [60].

Finally, mercurials have also been reported to significantly reduce the transcripts and protein abundance of AQP3, AQP4 and AQP7 in the gastrointestinal tracts of rats [95].

### 3.2. Zinc Ions

Zinc is one of the most abundant trace elements in the human body [96]. Many biochemical functions have been discovered for zinc cations, including three main roles: a structural role [97], a catalytic role [98] and a role in the maintenance of plasma membranes’ functions [99]. Zinc is known to interact with protein motifs called zinc fingers, which are found in many transcription factors. These motifs are characterized by cysteine and histidine residues positioned close to each other and forming the zinc fixation site [100]. Zinc has been shown to significantly increase the water permeability of *Xenopus laevis* oocytes expressing bovine AQP0 [101]. Moreover, the authors concluded that a zinc-mediated increase in water fluxes required positive cooperativity between sub-units associated with a Hill coefficient of 4 [101]. This result was further confirmed by a molecular dynamics study in which zinc fixation at the vicinity of human AQP0 cysteine 144 induced a significant increase in water permeability associated with a cooperative effect between monomers [102]. In this study, the putative zinc-binding site was predicted to be at the interface between sub-units. Upon zinc binding, two adjacent sub-units would hence be more tightly interacting with each other thanks to a salt bridge network between the two sub-units coordinated by zinc ions. This in turn resulted in an increase in the overall AQP0 tetrameric fold stability, which was associated with a re-positioning of R187 of the ar/R constriction sidechain within the pore lumen and higher water permeability [102]. Similar zinc-binding sites (near C145) and associated molecular mechanisms were also described for human AQP5 in the same study [102]. AQP5 is mainly expressed in secretory glands, where it plays a central role in fluid secretion [8,103]. Interestingly, another study has demonstrated that zinc supplementation could induce an increase in these glands’ secretory function [104]. However, mutagenesis studies indicate H40 and H122 to be necessary for zinc modulation of bovine AQP0 [101], while zinc-binding sites predicted through molecular dynamics did not incorporate these two histidines [102]. To explain these discrepancies, the authors of the molecular dynamics study formulated the hypothesis that H40 and H122 could be implicated in alternative ways. Histidine 40 could be involved in the accessibility of zinc cations to their binding site through the central pore, and histidine 122 could be involved in the transduction of zinc-binding signals to the ar/R constriction through loop C [102]. 

In an opposite manner, rat AQP4 reconstituted into proteoliposomes was shown to be transiently inhibited by zinc cations [105]. This inhibition is dependent on intra-cellular zinc (AQP4 was inserted in both directions into proteoliposomes) since no inhibition was observed for AQP4 expressed in *Xenopus laevis* oocytes [101]. Again, this result was comforted by molecular dynamics [102]. AQP4 possesses three intra-cellular cysteines: C87, C178 and C253. All three associated putative zinc-binding sites were tested through molecular dynamics, and only the C253 site was associated with a significant decrease in human AQP4 water permeability [102]. This result contrasted with the suppression of mercury inhibition in the AQP4 C178S mutant. It is, however, worth noting that the other cysteines were not mutated to compare with C178 [105]. According to the authors of the molecular dynamics study, the mutation of C178 to serine could also have induced small conformational re-arrangements locally. The C178S mutation could hence have hindered zinc binding through its impact on adjacent residue D179, which was involved in zinc stabilization with C253 [102]. Still, in the same molecular dynamics study, zinc fixation to human AQP2 intra-cellular C75 also induced a significant decrease in water permeability [102]. The associated molecular mechanism was similar between AQP4 and AQP2 but differed from the one explaining AQP0 and AQP5 water permeability increase. In this case, zinc binding did not increase the AQP tetrameric fold stability but rather impacted the orientation of the sub-unit dipole moment. Because the arginine of the ar/R sidechain is both positively charged and not stabilized by a salt bridge with a carboxylate residue, the authors postulated zinc fixation to orient this sidechain through long-distance electrostatic interactions [102]. 

Additionally, zinc was shown to alter transcripts abundance of plant AQPs in soybeans [106], barley [107] and pak choi [108]. In the study led on pak choi, the regulation of AQP genes differential expression was dose-dependent [108]. 

### 3.3. Cadmium Ions

As mentioned previously, cadmium was found in several structures of plant AQP *So*PIP2;1 [31,60] and human AQP2 [63]. This cation can be added for methodological purposes to increase the crystal quality [31]. Cadmium ions also induced a 70% water permeability loss in *At*PIP2;1 reconstituted in proteoliposomes [62]. Its toxicity could hence be partly explained by the interference with native calcium regulation of plant AQPs intra-cellular gating and AQP2 cellular trafficking (see calcium section). The same residues as for calcium-dependent intra-cellular gating E31 and H199 (H193 in *So*PIP2;1) were involved in cadmium sensitivity as well as loop B arginine124 [62].

### 3.4. Gold Ion Compounds

In the last decade, gold compounds have shown promising results as aquaglyceroporin inhibitors for the development of new therapies and imaging opportunities [109]. Indeed, more and more information has been piling up, indicating AQPs as elusive but interesting therapeutic targets in the fight against several cancers [25,26,109]. A new field of research has therefore emerged in the search for specific AQP inhibitors [110], among which are gold compounds. Several studies have pointed out C40 in AQP3 as the main residue responsible for gold compounds’ specific binding to the aquaglyceroporin [109,111,112,113,114,115,116]. Gold compounds were shown to induce a significant decrease in glycerol permeability of AQP3 through stopped-flow spectroscopy using human red blood cells. Through combined molecular and quantum dynamics, the authors detailed gold compounds’ interaction with C40 and highlighted a similar molecular mechanism as for the mercury inhibition of AQP3, i.e., upon gold compound binding, the arginine of the ar/R constriction sidechain undergoes a transconformation and occludes the pore [114]. Another AQGP (AQP7) has also been investigated for gold compound inhibitors. Gold compounds’ inhibition of AQP7 was significant and was evaluated by assessing both water and glycerol permeability in murine adipocyte cell line 3T3-L1 [117]. In silico docking highlighted pore-lining methionine 47 as a putative gold compound binding site for AQP7 [117]. Finally, gold particles have been shown to up-regulate AQP1 expression in mouse cerebral endothelium cell line bEnd.3 [118].

### 3.5. Silver Ions

Similarly to gold compounds, silver compounds were investigated with the aim of finding new AQP-specific inhibitors [119]. The authors tested the effect of silver nitrate on the permeability of the peribacteroid membrane (PBM) of soybean nodules, beetroot plasma membrane and human red blood cell membrane through a stopped-flow fluorimeter coupled with dynamic laser light scattering [119]. Each of the membranes is naturally enriched in one type of AQP: PBM is enriched in Nodulin-like integral protein NOD26 (a class of AQPs dedicated to ammonium uptake and specific to nitrogen-fixing plants [120]); the beetroot plasma membrane is enriched in plant strict AQPs (PIPs); the human red blood cell membrane is enriched in mammalian strict AQP1 and aquaglyceroporine AQP3. Silver was found to be the most efficient AQP inhibitor, regardless of the type of AQP when compared to other metallic cation compounds such as gold or mercury [119]. However, no mutagenesis or in silico modeling studies were carried out to study the molecular mechanisms involved. Silver nitrate also triggered AQP transcript increases in soybeans [106].

### 3.6. Copper Ions

Copper is an essential transition metal necessary for the function of several enzymes [121]. Copper sulfate was shown to significantly decrease water and glycerol permeability of human AQP3 but had no effect on human AQP4 or mouse AQP7 function [122]. AQP3 W128A, S152A and H241A mutants lost their sensitivity to copper, highlighting the role of these three residues in copper-mediated AQP3 inhibition. An in vitro study of copper-based AQP inhibitors displayed anti-tumorigenesis effects [123]. 

### 3.7. Nickel Ions

In a previous study, the same authors characterized the effect of nickel ions on human AQP3, AQP4 and AQP5 function [124]. Similarly, nickel ions induced significant water permeability inhibition in AQP3 but not in AQP4 or AQP5. Moreover, AQP3 was selectively inhibited by nickel and not by cadmium or zinc. Finally, the same residues as for copper (W128, S152 and H241) were shown to abolish nickel sensitivity when mutated in alanine [124]. Nickel ions also induce a 48% water permeability loss in *At*PIP2;1 reconstituted in proteoliposomes [62].

### 3.8. Lead Ions

Lead is another transition metal known for its high toxicity [125]. In the central nervous system, acute lead poisoning has been associated with astrocyte swelling [126]. The main AQP of the central nervous system, expressed in astrocytes, is AQP4 [127]. Through the over-expression of mouse AQP4 in an astrocyte cell line initially not expressing AQP4, Gunnarson et al. observed a significant increase in water permeability of these cells when cultivated with lead while no such effect was observed in untransfected cells or cells transfected with human AQP3 [128]. This lead effect seems to be mediated by calmodulin-dependant protein kinase II (CaMKII). Indeed, the inhibition of CaMKII abolished the lead-associated increase of water permeability. Moreover, the mutation to alanine of a serine of AQP4 consensus site for CaMKII phosphorylation (S111) abolished the lead effect on water permeability as well [128].

### 3.9. Manganese Ions

Manganese is a trace element necessary for the normal physiology of bacteria [129], animals [130,131] or plants [132]. The inhibition of water permeability through direct interaction with AQP has been demonstrated for plant AQP *At*PIP2;1 reconstituted in proteoliposomes [62]. This inhibition was similar to calcium’s and corresponded to a 60% loss of water permeability. Alanine mutants highlighted the role of H199 and, to a lesser extent, R124 and E31 in manganese-mediated gating of *At*PIP2;1 [62]. Manganese was also shown to interfere with AQP2 and AQP4 trafficking. Manganese treatment induced AQP2 internalization and intra-cellular accumulation in cultured cells [133]. On the contrary, the treatment of cultured astrocytes with manganese increased AQP4 proteins in the plasma membrane [134]. 

### 3.10. Iron Ions

Iron is essential for human physiology [135]. However, its over-accumulation in the brain can lead to brain edema and neurodegenerative diseases such as Alzheimer’s disease and Parkinson’s disease [136,137]. The upregulation of AQP4 and AQP9 protein levels was observed in cultured astrocytes treated with iron [138]. In another study, cultured astrocytes were exposed to different concentrations of iron ions [139]. Iron-induced astrocyte swelling and death happened in a dose- and time-dependent manner. AQP4 mRNA and protein levels assessed by RT-qPCR and immunofluorescence staining were also significantly increased by iron treatment [139]. In the same study, it was observed that the inhibition of oxidative stress and Mitogen-activated protein kinases (MAPK) both reduced iron-induced astrocyte death and AQP4 upregulation [139]. Iron effect on AQP4 expression is hence probably mediated by MAPKs. Furthermore, immunohistochemical analysis and real-time quantitative polymerase chain reaction of AQP4 in rat brains showed an association between iron overload, AQP4 expression increase and intracerebral hemorrhage [140,141]. Moreover, hydrocephalus was attenuated by an AQP4 inhibitor (2-(nicotinamide)-1,3,4-thiadiazole: TGN-20) [141]. 

## 4. Alkaline Metals

### 4.1. Lithium Ions

Lithium has been widely used to treat bipolar disorders [142]. Chronic lithium treatment is associated with the development of nephrogenic diabetes insipidus (NDI) [143], which is associated with AQP2 downregulation [144,145]. Several studies have demonstrated a decrease in AQP2 gene expression and protein abundance after lithium exposure [144,145,146,147,148,149,150]. The protein abundance of AQP3 was also significantly reduced after the lithium treatment of rats, while AQP1 was not affected [144].

### 4.2. Sodium Ions

Sodium ions have also been shown to significantly impact transcript and protein levels of AQP2 in rats and humans. Except for one study in which Sprague–Dawley rats subjected to a high NaCl diet displayed a lower abundance of AQP2 transcripts compared to basal NaCl diet [151], all the other studies associated high NaCl diet or treatment with increased AQP2 transcripts or protein abundance in vivo [152,153,154,155] or in vitro [156]. Recently, two molecular dynamics studies on the regulation of human AQP2 highlighted a putative direct modulation of AQP2 water permeability by sodium ions [157,158]. The authors observed a preferential interaction of sodium ions with the carboxylates of extra-cellular loop C and loop E (E106, D111, D115, D199 and D200) compared to potassium ions. This interaction interfered with the ability of these carboxylates to establish salt bridges with other charged residues and on the overall stability of loop C and was also associated with ar/R constriction arginine 187 conformational changes and hence water permeability changes as well [157,158]. More research is however needed to assess if AQP2 is directly modulated by sodium ions or not. Supporting a specific regulatory role of sodium in AQP regulation, a study led on the halophytic grass *Puccinellia nuttalliana* highlighted an increase in root cell hydraulic conductivity after sodium and not after potassium treatment, which was abolished by mercury [159].

## 5. Discussion

Since the discovery of AQPs by Peter Agre in 1992 [3], researchers have been constantly intrigued by the functional complexity underlying this family of channel proteins [20,21]. Initially conceptualized as water channels only, the first challenge was to understand how they could nonetheless exclude protons [28]. It was then discovered over the years that they could also accommodate other small polar molecules [160], reactive oxygen species or even ions for a few of them [161]. On top of the traditional regulation of their gene expression, many other factors have been shown to significantly modulate AQPs’ function: sub-cellular trafficking [53,162], membrane composition [34], protein-protein interactions [35], allosteric mechanisms between sub-units of the same tetramer [163] or gating [164]. In this review, we highlighted the significant contribution of cations as well, which interpose in every level of the AQPs regulations previously mentioned. All of the modulation of AQPs by cations are summarized in Table 1, all putative cations-binding-sites are indicated on an archetypal AQP fold in Figure 3 and all of the residues involved in these modulations are indicated on a multiple sequence alignment of human AQPs, *So*PIP2;1 and *Ec*AQPZ in Supplementary Figure S1.

While it appears clearly that cations are relevant physiological regulators of AQPs in all types of organisms, a global understanding of their modes of action is lacking. The initially proposed steric inhibition of water permeability by mercury bound to pore lining cysteine residues is for sure insufficient or too restrictive to explain all types of cation-associated regulations of AQPs. Indeed, there is for instance no clear explanation for allosteric modulations not associated with the targeting of pore-lining residues by cations nor for mercury mode of action in plant AQPs which does not seem to involve cysteines at all. However, from this reviewing work, a few tendencies can be extracted: (i) Most of the described cations-binding-sites are associated with AQP inhibition except for the one located on the helix 4 cytoplasmic end, at the interface between two sub-units (see Figure 3). Indeed, three different studies indicate the fixation of zinc or mercury to this cations-binding site as a trigger for AQP activation. Moreover, this phenomenon is associated with cooperativity between sub-units. (ii) In most cases, cations act upon several layers of regulation, e.g., calcium has been associated with gene expression modulation, sub-cellular trafficking, protein–protein interactions and the gating of AQP2. (iii) Cysteines are far from being the only residues involved in cations binding in AQPs. Other residues such as serines, threonines, glutamines, methionines and carboxylates can also be found in AQPs cations binding sites. (iv) Most of the molecular mechanisms mentioned in this review eventually lead to the fine-tuning of the position of the sidechain of the arginine of the ar/R constriction inside the pore lumen. 

The arginine of the ar/R constriction is among the most conserved residues of the AQP fold and is also known to be a determinant of the solute selectivity of the pore. Because of their positively charged sidechains, arginine can act as voltage sensors in voltage-gated channels [166,167]. Considering the perturbations of the protein electrostatic field induced by cation binding, one could hypothesize a similar role for the arginine of the ar/R constriction in AQPs. Several studies have demonstrated the possible modulations of AQPs through the application of external electric fields in silico [168,169,170,171,172,173,174]. While other pore-lining residues such as histidines were involved, the ar/R constriction arginine was indeed implicated in external electric field sensing [168,171,172,173,174]. Other studies highlighted the modulation of AQPs’ water fluxes by non-physiological membrane potentials through all-atom molecular dynamics [175,176]. Once again, the ar/R arginine played a central role. Interestingly, high but realistic concentrations of KCl ions could also induce the same type of modulation [176]. Better understanding how charge repartition at different locations of the AQP fold could impact ar/R constriction arginine conformation inside the pore lumen could hence be a way to propose a unifying molecular mechanism of AQP modulation by cations. To conclude, it appears that cations play a significant role in the regulation of AQPs’ function. Moreover, on top of better understanding this regulatory network, more general theoretical knowledge about AQPs and ion channels function could also be gathered from this field of study.

## Figures and Tables

**Table 1:** Known modulations of AQPs by cations. All of the modulations of AQPs by cations discussed in the current review are summarized. The corresponding references are indicated under brackets.

Ion Name	Name of the Regulated AQP	Direct Interaction with AQP	Water Channel Function	Residues Involved in Binding	Residues Involved in Molecular Mechanism	Transcripts and/or Protein Abundance
Calcium	*So*PIP2;1	yes	inhibition [31]	D28 and E31 [31] T183 and A267 [60]	D28, E31, R190, R191 and H193 [31,57]	Unknown
Calcium	*So*PIP2;1	No	Activation	None	S115, S188 and S274 [31,58,59]	unknown
Calcium	*At*PIP2;1	Yes	Inhibition	E31, H199 [62]	E31, H199 and R124 [62]	unknown
Calcium	Human AQP2	yes	Unknown	Q57, E155 [56,63]	Unknown	increase [50,165]
Calcium	Ovine AQP0 Human AQP0	No	Inhibition	None	L227, L234, A240 and R156 (CaM binding) [41,55] S229, S231 and S235 (phosphorylation sites) [46] R187, Y149, Y23 (water permeability modulation) [45,54]	unknown
Calcium	Human AQP4	No	Unknown	None	Residues 6-31 in AQP4-M1 residues 256-280 in AQP4-M1 (CaM binding) [49]	unknown
Calcium	Mouse AQP6 rat AQP6 human AQP6	No	Unknown	None	L15, L19, A22 and F28 in human AQP6 [48]	unknown
Magnesium	Human AQP3	Unknown	Unknown	Unknown	Unknown	increase [67,68,69]
Magnesium	Rat AQP4 human AQP4	Unknown	Unknown	Unknown	Unknown	No effect or decrease [70,71,72,73]
Mercury	E. coli AQPZ	yes	Inhibition	C20 [82]	C20 [82] R189 [92]	unknown
Mercury	Human AQP3	yes	Inhibition	C40	F63, R218 [86]	Decrease (rat AQP3) [95]
Mercury	Human AQP1 bovine AQP1	yes	Inhibition	Human: C189 [80] bovine: C191 [88]	For bovine AQP1: R197, H182, F58, I193, G192, C191, G190, E144 [88]	Unknown
Mercury	Human AQP2	yes	Inhibition	C181 [87]	Unknown	unknown
Mercury	rat AQP4	yes	Inhibition	C178 [83]	Unknown	Decrease [95]
Mercury	Rat AQP6	yes	Activation	C155 and C190 [85,90]	R196 [92] and M160 [91]	No effect
Mercury	*At*TIPs	Yes	Inhibition	C116 or C118 [93]	C116 or C118 [93]	unknown
Mercury	Rat AQP7	Unknown	Unknown	Unknown	Unknown	decrease [95]
Zinc	bovine AQP0 human AQP0	yes	Activation	H40 and H122 [101] C144, T54, Q140 [102]	R187 [102]	unknown
Zinc	Human AQP5	yes	Activation	C145, S149, S164, S168, T55, Q58 [102]	Human: R187 [102]	increase [104]
Zinc	Rat AQP4 Human AQP4	yes	Inhibition	Rat: C178 [105] human: C253, D179, H90 [102]	Human: R216 [102]	unknown
Zinc	Human AQP2	yes	Inhibition	C75 [102]	R216 [102]	unknown
Zinc	Soybean AQPs	Unknown	Unknown	Unknown	Unknown	No effect or increase [106]
Zinc	Barley AQPs (HvPIP1;3, HvPIP2;4 and HvPIP2;5)	Unknown	Unknown	Unknown	Unknown	decrease [107]
Zinc	Pak choi AQPs (PIP1 isoforms)	unknown	Unknown	Unknown	Unknown	increase [108]
Zinc	Human AQP3	No	No effect [124]	None	None	unknown
Cadmium	Human AQP2	Yes	Unknown	Q57, E155 [63]	Unknown	unknown
Cadmium	*So*PIP2;1	Yes	Inhibition	D28 and E31 [31] T183 and A267 [60]	D28, E31, R190, R191 and H193 [31,57]	unknown
Cadmium	*At*PIP2;1	Yes	Inhibition	E31 and H199 [62]	E31, H199 and R124 [62]	unknown
Cadmium	Human AQP3	No	No effect [124]	None	None	Unknown
Gold	Human AQP3	yes	Inhibition	C40 [114]	R218 [114]	unknown
Gold	Human AQP7	yes	Inhibition	Met47 [117]	unknown	Unknown
Gold	mouse AQP1	Unknown	Unknown	Unknown	Unknown	increase [118]
Silver	Soybean NOD26	Unknown	inhibition [119]	Unknown	Unknown	unknown
Silver	Beet root PIPs	Unknown	inhibition [119]	Unknown	Unknown	unknown
Silver	Human AQP1 and human AQP3	Unknown	Inhibition [119]	Unknown	Unknown	unknown
Silver	Soybean AQPs	Unknown	Unknown	Unknown	Unknown	No effect or increase [106]
Copper	Human AQP3	Unknown	inhibition	unknown	W128, S152, H241 [122]	Unknown
Copper	Human AQP4	no	No effect [122]	None	none	unknown
Copper	Mouse AQP7	no	No effect [122]	None	none	Unknown
Nickel	Human AQP3	Unknown	Inhibition	unknown	W128, S152, H241 [124]	Unknown
Nickel	Human AQP4	no	No effect [124]	none	none	unknown
Nickel	Human AQP5	no	No effect [124]	None	None	unknown
Nickel	*At*PIP2;1	Yes	Inhibition [62]	Unknown	unknown	unknown
Lead	Mouse AQP4	unknown	Activation	Unknown	S111 [128]	No effect [128]
Lead	Human AQP3	No	No effect [128]	None	None	unknown
Manganese	*At*PIP2;1	Yes	Inhibition	E31 and H199 [62]	E31, H199 and R124 [62]	unknown
Manganese	Rat AQP2	Unknown	Inhibition [133]	Unknown	Unknown	unknown
Manganese	Rat AQP4	Unknown	Activation [134]	Unknown	Unknown	No effect
Iron	Mouse AQP4 rat AQP4	Unknown	Unknown	Unknown	Unknown	increase [138,139,140,141]
Iron	Mouse AQP9	Unknown	Unknown	Unknown	Unknown	increase [138]
Lithium	Human AQP2 [146] rat AQP2 [144,145,148,149] mouse AQP2 [147,150]	Unknown	Unknown	Unknown	Unknown	decrease [144,145,146,147,148,149,150]
Lithium	Rat AQP3	Unknown	Unknown	Unknown	Unknown	decrease [144]
Lithium	Rat AQP1	Unknown	Unknown	Unknown	Unknown	No effect [144]
Sodium	Rat AQP2 [151,154,156] human AQP2 [152,153,155]	yes [157]	Unknown	E106, D111, D115, D199 and D200 in human AQP2 [157]	Unknown	Conflicting results: decrease [151] increase [152,153,154,155,156]

## Supplementary Materials

The following supporting information can be downloaded at: https://www.mdpi.com/article/10.3390/cimb46080470/s1.
