# Supplementary material for: Aquaporin Modulation by Cations, a Review
Source: Curr Issues Mol Biol. 2024 Jul 24;46(8):7955–75. doi: 10.3390/cimb46080470 (PMC11353066; doi:10.3390/cimb46080470)
Supplement: Supplementary file 1 [file cimb-46-00470-s001.zip › cimb-3117623-supplementary.pdf]

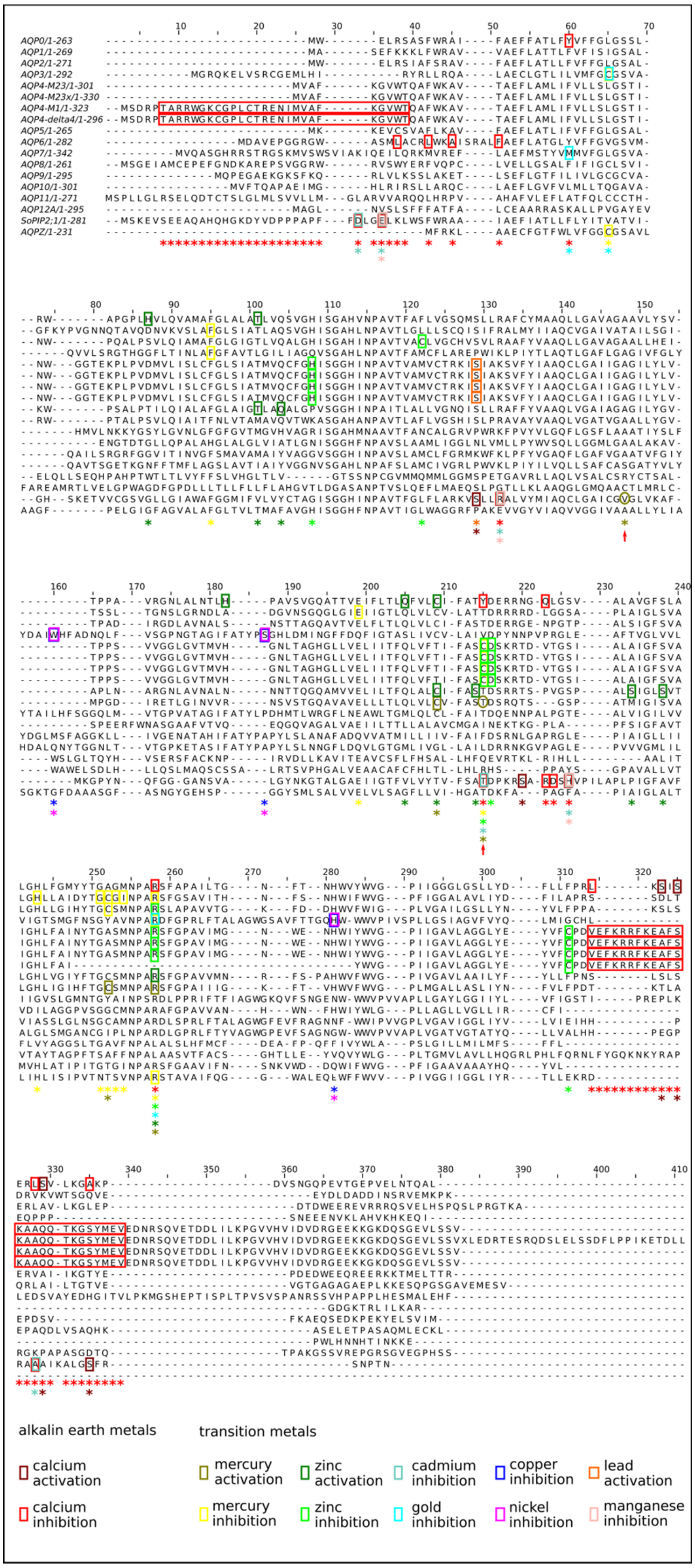

Figure S1. AQPs modulation by cations. Multiple alignment of human AQPs, spinach *So*PIP2;1 and E. coli AQPZ. All residues involved in AQP activation or inhibition and discussed in the current review

are indicated by colored boxes on the corresponding positions on the alignment. For clarity purposes, stars of the corresponding colors have been added under the alignment. Two residues indicated by circles correspond to amino acids of different nature in the original sequence: Valine 134 in *So*PIP2;1 is replaced by a cysteine in the Arabidopsis thaliana AQPs modulated by mercury [1]; Threonine 163 in human AQP6 is replaced by a methionine in rat AQP6 modulated by mercury [2]. The positions in the alignment for these two residues are indicated by red arrows.

References

1. Daniels, M.J.; Chaumont, F.; Mirkov, T.E.; Chrispeels, M.J. Characterization of a New Vacuolar Membrane Aquaporin Sensitive to Mercury at a Unique Site. *Plant Cell* **1996**, *8*, 587–599, doi:10.1105/tpc.8.4.587.

2. Ma, S.; Xie, H.; Yu, K.; Yang, J. Mechanism of Unusual AQP6 Activation by Mercury Binding to a Pore-External Residue C155. *Biochemical and Biophysical Research Communications* **2022**, *618*, 1–7, doi:10.1016/j.bbrc.2022.06.025.
